# Use of fluorescent oligonucleotide probes for differentiation between *Paracoccidioides brasiliensis* and *Paracoccidioides lutzii* in yeast and mycelial phase

**DOI:** 10.1590/0074-02760160374

**Published:** 2017-02

**Authors:** Thales Domingos Arantes, Raquel Cordeiro Theodoro, Marcus de Melo Teixeira, Eduardo Bagagli

**Affiliations:** 1 Universidade Estadual Paulista, Instituto de Biociências de Botucatu, Departamento de Microbiologia e Imunologia, Botucatu, SP, Brasil; 2 Universidade Federal do Rio Grande do Norte, Centro de Biociências, Instituto de Medicina Tropical, Programa de Pós-Graduação em Bioquímica, Campus Universitário Lagoa Nova, Natal, RN, Brasil; 3 Universidade Federal do Rio Grande do Norte, Centro de Biociências, Departamento de Biologia Celular e Genética, Natal, RN, Brasil; 4 Northern Arizona Center for Valley Fever Research, Translational Genomics Research Institute - Tgen North, Phoenix, AZ, US

**Keywords:** paracoccidioidomycosis, fluorescence *in situ* hybridisation, tyramide signal amplification, *Paracoccidioides* spp

## Abstract

**BACKGROUND:**

Fluorescence *in situ* hybridisation (FISH) associated with Tyramide Signal Amplification (TSA) using oligonucleotides labeled with non-radioactive fluorophores is a promising technique for detection and differentiation of fungal species in environmental or clinical samples, being suitable for microorganisms which are difficult or even impossible to culture.

**OBJECTIVE:**

In this study, we aimed to standardise an *in situ* hybridisation technique for the differentiation between the pathogenic species *Paracoccidioides brasiliensis* and *Paracoccidioides lutzii*, by using species-specific DNA probes targeting the *internal transcribed spacer*-1 (ITS-1) of the rRNA gene.

**METHODS:**

Yeast and mycelial phase of each *Paracoccidioides* species, were tested by two different detection/differentiation techniques: TSA-FISH for *P. brasiliensis* with HRP (Horseradish Peroxidase) linked to the probe 5’ end; and FISH for *P. lutzii* with the fluorophore TEXAS RED-X^®^ also linked to the probe 5’ end. After testing different protocols, the optimised procedure for both techniques was accomplished without cross-positivity with other pathogenic fungi.

**FINDINGS:**

The *in silico* and in vitro tests show no reaction with controls, like *Candida* and *Cryptococcus* (*in silico*) and *Histoplasma capsulatum* and *Aspergillus* spp. (in vitro). For both phases (mycelial and yeast) the *in situ* hybridisation showed dots of hybridisation, with no cross-reaction between them, with a lower signal for Texas Red probe than HRP-TSA probe. The dots of hybridisation was confirmed with genetic material marked with 4’,6-diamidino-2-phenylindole (DAPI), visualised in a different filter (WU) on fluorescent microscopic.

**MAIN CONCLUSION:**

Our results indicated that TSA-FISH and/or FISH are suitable for *in situ* detection and differentiation of *Paracoccidioides* species. This approach has the potential for future application in clinical samples for the improvement of paracoccidioidomycosis patients prognosis.

*Paracoccidioides* species present a relatively slow growth in culture, which difficult their clinical and environmental isolation by direct culture. Therefore, many efforts have been concentrated on molecular detection techniques (Theodoro et al. 2005, Terçarioli et al. 2007, Arantes et al. 2013) such as polymerase chain reaction (PCR) and its variations, which have been largely used for *Paracoccidioides* spp. detection in several environmental samples (soil, plants and animal tissues) (Bagagli et al. 1998, Corredor et al. 2005, Theodoro et al. 2005, Richini-Pereira et al. 2008, Taylor 2011, Arantes et al. 2013).

Clinically, the reference laboratorial method for diagnosis of *Paracoccidioides* spp. is the direct visualisation of the pathogen in tissue or its isolation in culture (Mendes & Shikanai-Yasuda 2003, Shikanai-Yasuda et al. 2006), but in cases of few infective fungal cells or uncultivable material, molecular techniques such as *in situ* detection of specific target DNA could be a promising choice (Perlin & Zhao 2009 , Ampel 2010 , Koepsell et al. 2012 , Litvintseva et al. 2015) for disease diagnosis because it acts directly in the biological sample (Nakagome et al. 1991 , Moter & Gobel 2000 , Liehr 2009). This method has opened a new perspective not only for environmental research of *Paracoccidioides* species (de Brito 1999 , Arantes et al. 2016), but also for their detection in patient’s tissues. This might also be an interesting strategy mainly for uncultivable fungi, such as *Lacazia loboi* another systemic pathogen from Ajellomycetacea family (Lacaz et al. 2002).

*The fluorescence in situ hybridisation* - Fluorescence *in situ* hybridisation (FISH) technique is usually associated with tyramide signal amplification (TSA), which is based on the ability of peroxidase (HRP), in the presence of low concentrations of H_2_O_2_, to convert the labeled tyramide in a substrate containing highly reactive oxidised free radicals that can covalently bind to tyrosine residues at or near to the HRP (Adams 1992). This technique is an alternative for research with environmental samples, because it exponentially increases the fluorescence signal. For instance, this technique was effective in the detection of an entire new phylum within the fungal kingdom, named Cryptomycota (Jones et al. 2011).

*In situ* hybridisation (ISH) has already been carried out for *Paracoccidioides* detection in patient’s oral biopsies, targeting the 28S rRNA coding gene with a 14 nucleotide probe (de Brito 1999). However, despite of the interesting results, when compared to the large current available database for fungal rRNA sequences, this probe cannot distinguish between *P. brasiliensis* and *P. lutzii* and also shows complementarity to 28S gene from many other fungal species. This can be explained by the high conservative nature of the 28S rRNA coding gene (White et al. 1990). So, despite of the high sensitivity of this probe, whose target is a multi-copy gene, its specificity is compromised.

In this work we purposed the use of probes whose target, the internal transcribed spacer 1 (ITS1), from the rRNA coding sequence, is multi-copy and also variable in the fungal genomes, being conserved within *P. brasiliensis* and also within *P. lutzii.* The specificity of this probes was tested *in silico* and in vitro, mainly in the yeast phase of these fungi. We also described the detailed standardisation of the *in situ* hybridisation protocol for *Paracoccidioides* differentiation in mycelial (saprophyte) and also in yeast (parasitic) phase, which we have already successfully applied for ecological studies on the geographical distribution of the *Paracoccidioides* species (Arantes et al. 2016), and now the propose of the use of *in situ* hybridisation for yeast phase to differentiate both pathogens directly on clinical samples.

## MATERIALS AND METHODS

*In silico analysis for probes design* - The rRNA coding gene, more specifically the ITS-1 sequence (White et al. 1990), was used for the differential probe design as previously described for environmental aerosol samples (Arantes et al. 2016). In addition, the partial rRNA sequences from different *Paracoccidioides* genotypes (Teixeira et al. 2009, Arantes et al. 2013) were aligned in order to select conserved regions within species, but polymorphic between them, so that one probe hybridises to the species complex *P. brasiliensis* and the other to *P. lutzii*. GenBank sequences used were EU870314; EU870315; AY631235; EU118561; EU118560; EU118548; EU118554; EU118553; EU118549; EU118546; EU118547; EU118545; EU118543; EU118542 (*P. brasiliensis*) and EU870298; EU870303; EU870306; EU870309; EU870310; EU870311; AF092903; EU870299 (*P. lutzii*). In order to check the specificity of the probes for *Paracoccidioides* genus, ITS1-5.8S-ITS2 sequences of phylogenetically nearby fungi were also used in the *in silico* analysis, such as *Histoplasma capsulatum* and *Emmonsia* sp., access number AF129547 and AF038322 respectively, from Ajellomycetaceae family, as well as sequences of common fungal pathogens in clinical specimens, as *Candida albicans* (EF192231), *Candida parapsilosis* (EF68035), *Candida tropicalis* (EF190225), *Candida glabrata* (KX130866) and *Candida metapsilosis* (EF190228), *Cryptococcus neoformans* (KT585710; KT958228; KT958227) and *Cryptococcus gattii* (FJ534878; FJ534877; KC171355). The sequences were aligned in MEGA 6.0 software by using the ClustalW algorithm (Thompson et al. 1994, Tamura et al. 2013). Thirty-eight ITS sequences from *P. brasiliensis*, 19 from *P. lutzii*, two from *Histoplasma capsulatum* and two from *Emmonsia* sp. were used. The designed probes were submitted to the similarity analysis on NCBI site database by using the Blastn tool (Altschul et al. 1990) to check and confirm their specificity. The *in silico* analysis was used to exclude any possible cross hybridisation to other fungal species.

*Fungal samples for standardisation of FISH and TSA-FISH methods* - Three *P. brasiliensis* isolates (T16B1, Pb192 and T15LN1) and three *P. lutzii* (Pb01, Pb66 and PbEE) were used as positive controls for standardising hybridisation and for specificity test (cross-hybridisation between then). Isolates were obtained from the mycology collections of the Fungal Biology Lab (Department of Microbiology and Immunology, Biosciences Institute, UNESP, Botucatu, SP) and Fungal Molecular Biology Lab (University of Brasilia - UnB, DF). These isolates are from clinical (Pb192, Pb01, Pb66 and PbEE) and armadillo samples (T16B1 and T15LN1), and have been previously identified as *P. brasiliensis* or *P. lutzii* (Arantes et al. 2013).

For specificity test, the fungi *Histoplasma capsulatum*, *Aspergillus flavus*, *Aspergillus fumigatus* and *Trichophyton mentagrophytes* (Lacaz et al. 2002) are tested with *Paracoccidioides* spp. probes. They were used because have a higher positivity rate in clinical samples, in addition to being possible interferences as hybridisation probes in mycological laboratories and as previously described (Arantes et al. 2016) in environmental samples. Fungi were maintained on agar Sabouraud petri plates until their use in experiments, with incubation time and temperature variables for each genus and/or species.

*FISH and TSA-FISH methods* - Reagent preparation for TSA-FISH method was adapted from the TSA PerkinElmer^®^ commercial kit and from the Sampling-Protocol Analysis: Parasitic-Host Dynamics Study, Nautset Marsh (Salt Pond and Mill Pond) and protocol described in Biegala et al. (2002). To colonies of mycelial phase, a fragment of 4mm^2^ was removed and added to the fixation solution, for the yeast phase, two calibrated handles (50 µL) were added to the fixative solution. The volume of fixing solution used in both cases was 3.0 mL.

For FISH and TSA-FISH fungal cultures (yeast and mycelia) were inactivated in fixation solution (4% paraformaldehyde plus 0.1 M phosphate buffer) for maintenance of their structure and genetic material. A sequence of 50%, 80% and 100% ethanol solutions were used to remove the fixation solution and dehydrate cells for posterior probe absorption in the hybridisation step. After dehydration, 10 mL of pre-hybridisation buffer [2.0 mL of ultra pure H_2_O; 4.0 mL of 40% Formamide; 1.8 mL of 5M NaCl; 200 μL of 1M Tris (pH 7.5); 100 μL of 1% SDS; 2 mL of 10% Buffer Blocking Agent] were added to the samples for stabilising and improving their permeability by differences in osmotic pressure. After this first step, the cells were hybridised with probes prepared in hybridisation buffer [2.85 mL MilliQ H20; 4.0 mL deionised formamide (40%); 1.8 mL NaCl 5M; 200 µL Tris 1M (pH 7.5); 100 µL 1% SDS; 2.0 mL Blocking buffer 10%] at a final concentration of 50 ng/μL. After 16-17 hours of incubation at 42ºC, the slides with fungal controls were washed with 50 mL of Washing Buffer [47.54 mL of ultra-pure H_2_O; 460 μL of 5M NaCl, 500 μL of 0.5M EDTA, 500 μL of 1% SDS and 1 mL of 1M Tris (pH 7.5)] for removal of non-specific binding probes. After washing, the slides were stabilised with 250 mL of TNT buffer [217.315 mL of ultra-pure H_2_O; 25 mL of 1M Tris (pH 7.5); 7.5 mL of 5M NaCl and 0.185 mL of Tween 20].

After equilibrating and washing the slides with TNT buffer, for TSA-FISH protocol, 30 μL of TSA solution from the commercial kit (TSA Plus PerkinElmer^®^) were added onto each slide, which were then incubated for 30 min in a dark humid chamber at room temperature. Then, the slides were washed again and dried at room temperature, following the addition of DAPI (4’,6-diamidino-2-phenylindole), a fluorescent dye that binds to DNA, mainly in A-T regions. We used DAPI in fluorescence microscopy tests to observe the DNA regions with integrity of cellular structures and to mark the genetic material and indicate the real dots of probe hybridisation, for distinguishing between the points of intracellular accumulation of the probes and actual hybridisation of the same. The slides were covered with a cover slip and observed under a fluorescence microscope. For FISH method, after addition of TNT buffer, the steps were the same applied for TSA-FISH slides but without addition of TSA solution.

## RESULTS

**Fig. 1:**
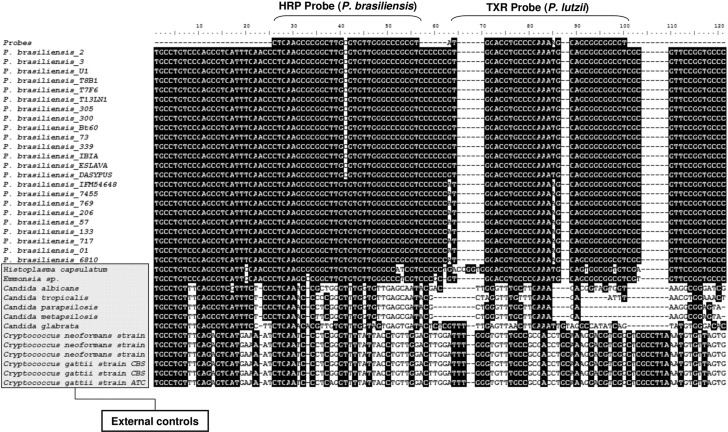
: *in silico* inferred probe specificity for *in situ* hybridisation of *Paracoccidioides brasiliensis* and *P. lutzii.* Aligned sequences of the internal transcribed spacer 1 (ITS1) rRNA region of *Paracoccidioides* spp., *Histoplasma capsulatum* and *Emmonsia* sp. used for the *in silico* tests of probe designing for *in situ* hybridisation of *P. brasiliensis* and *P. lutzii*, with fluorophores HRP + Fluorescein and Texas-Red, respectively. The design of specific probes was based on the divergences between the *Paracoccidioides* species and between the *Paracoccidioides* genus and other Ajellomycetacea species (*H. capsulatum* and *Emmonsia* spp), and other pathogenic yeasts like *Cryptococcus* sp. (Tremellaceae) and *Candida* spp. (Saccharomycetaceae).

*In silico analysis* - Probes were designed to target the ITS1 region of rRNA in *P. brasiliensis* and *P. lutzii*. The ITS probe/HRP for *P. brasiliensis* distinguishes it from *P. lutzii* due to the C/T SNP (Single Nucleotide Polymorphism) and the ITS probe/Texas Red for *P. lutzii* distinguishes it from *P. brasiliensis* due the G/A and T/A SNPs. Both probes are specific for *Paracoccidioides,* and therefore, not complementary to the ITS1 from *Histoplasma*, *Emmonsia*, *Candida* or *Cryptococcus* as demonstrated by the sequence alignment (Fig. 1)*.* The *in silico* analysis of probes performed on the NCBI database using the *Blastn* tool showed no cross-reactivity with other fungi.

*FISH for P. lutzii and TSA-FISH for P. brasiliensis mycelia and yeast cells* - The Texas-Red probe, specific for *P. lutzii*, successfully hybridised with mycelial cells of the Pb01, Pb66 and PbEE isolates (Supplementary data, Fig. 1A, C, E, G), while the HRP probe specific for *P. brasiliensis* hybridised mycelial cells of T16B1, T15LN9 and Pb192 (Supplementary data, Fig. 1I, K, M, O). The genetic material was labeled with DAPI as positive control for the nuclear hybridisation (Supplementary data, Fig. 1B, D, F, H, J, L, N, P).

**Fig. 2:**
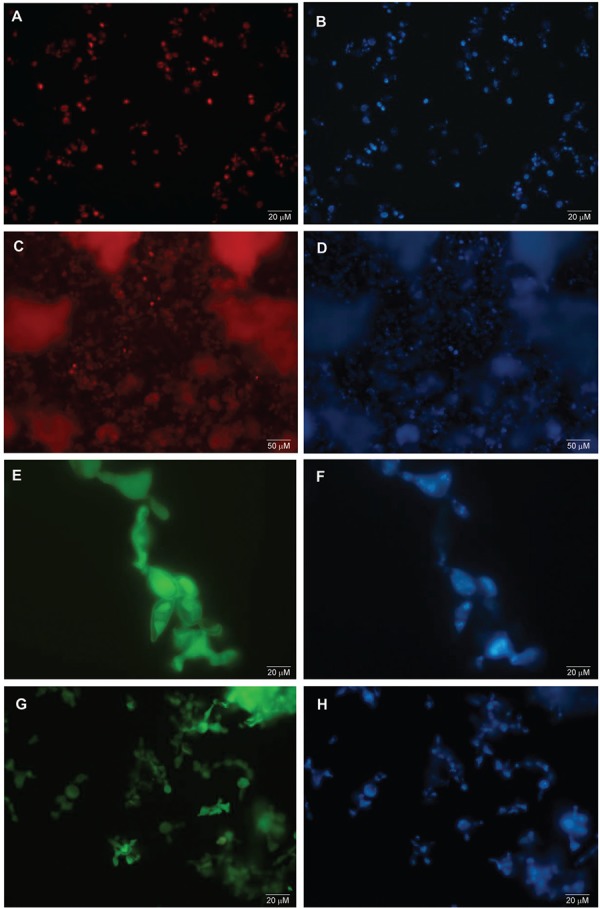
: *Paracoccidioides* spp. yeast cells hybridisation by FISH and TSA-FISH techniques. (A, C) Yeasts of *P. lutzii* isolate Pb01 hybridised with Texas-Red probe by FISH technique; (B, D) yeasts of *P. lutzii* stained with DAPI for genetic material visualisation in cellular structures; (E, G) yeasts of *P. brasiliensis* isolate T16B1 hybridised with HRP-Fluorescein probe by TSA-FISH technique; (F, H) yeasts of *P. brasiliensis* stained with DAPI for genetic material visualisation in cellular structures. (Magnification 400x and 1000x).

**Fig. 3:**
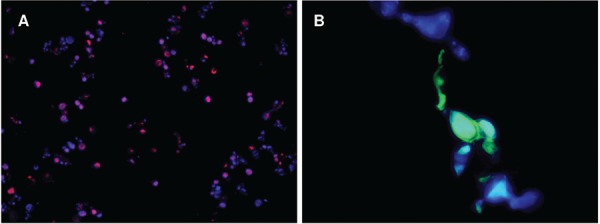
: merged images of the yeast hybridisation with *Paracoccidioides lutzii* and *P. brasiliensis* specific probes. (A) Yeasts of *P. lutzii* hybridised with TEXAS-Red probe indicated by intracellular red dots (400x); (B) yeasts of *P. brasiliensis* hybridised with HRP-TSA probe indicated by intracellular green dots (1000x); (A, B) cellular walls can be visualised by structures marked in blue colour.

Similarly, the species-specific probes Texas-Red and HRP-TSA hybridise to the yeast phase of *P. lutzii* (Fig. 2A-D), and *P. brasiliensis* (Fig. 2E-H), respectively. Some of these yeast cells hybridisation images for *P. lutzii* (Fig. 3A) and *P. brasiliensis* (Fig. 3B) were merged to the corresponding DAPI images obtained under the WU filter, using Image J software (Schineider et al. 2012) for a better visualisation of the hybridisation dots in yeast cells.

**Fig. 4:**
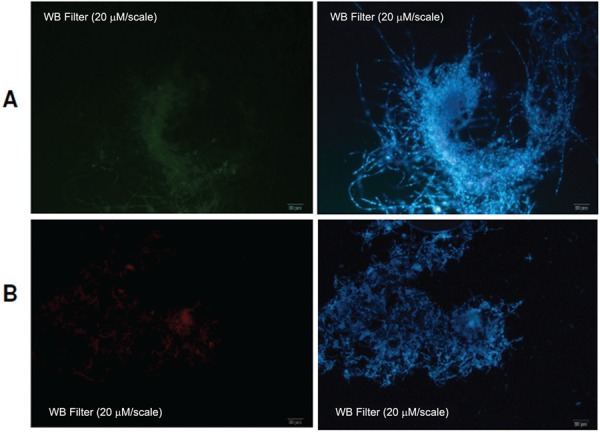
: test for cross-hybridisation of ITS HRP and ITS Texas-Red probes against *Paracoccidioides lutzii* and*. P. brasiliensis* respectively. (A) *P. lutzii* isolate Pb01 (400x), subjected to ITS HRP probe by TSA-FISH technique. No hybridisation was observed; (B) *P. brasiliensis* isolate T16B1 (400x), subjected to ITS Texas Red probe by FISH technique. No hybridisation was observed. For both isolates genetic material was visualised with DAPI (blue) under WU filter in a fluorescence microscope.

*Specificity and sensitivity in vitro tests* - There was no cross-hybridisation of Texas Red-probe (used in FISH for *P. lutzii* detection) or the HRP-probe (used in TSA-FISH for *P. brasiliensis)* to *P. brasiliensis* and *P. lutzii* isolates, respectively (Fig. 4). In Fig. 4A-B, we can observe the natural fluorescence of the fungal cells in a few points, due to accumulation of probes and/or fluorophores without signal emission points for hybridised probes. Also, no cross-reaction was observed for each probe against *H. capsulatum* (Arantes et al. 2016) or the other fungi tested, such as *Aspergillus flavus*, *A. fumigatus* and the dermatophyte *T. mentagrophytes* (Supplementary data, Fig. 2). Twenty four slides were prepared for this test (six for each isolate), and after observation under fluorescence microscope, no hybridisation was visualised for each probe, proving their specificity for *Paracoccidioides* spp. detection. However, retention of Texas Red probe within hyphae cells was detected on a single slide of *A. flavus* (Supplementary data, Fig. 2).

## DISCUSSION

In this study we modified existing protocols described in literature and commercial kits in order to use HRP and Texas-Red labeled oligonucleotides probes for identification of *Paracoccidioides* spp. cells in mycelial phase, as previously described (Arantes et al. 2016) and mainly in yeast phase, as a new approach.

During the optimisation of FISH (with Texas-Red probe) and TSA-FISH (with HRP-Fluorescein probe) techniques, a final protocol were adapted for satisfactory differentiation between *Paracoccidioides* species. Both probes were hybridised to their targets and showed no cross-reactivity within the genus and between *Paracoccidioides* spp. and other fungal species according to the *in silico* and in vitro analysis performed. For the probes’ specificity test against other fungi (Supplementary data, Fig. 2), we visualised some dots of probes signal, which were considered points of probes accumulation, mainly due to non-removal of not hybridised probes during the washing steps of the fungal cells, which were confirmed due to non-genetic material stained with DAPI inside these cell, when viewed with WU filter. So, we attributed this result to the retention of crystallised probe, even after the washing steps that follow the hybridisation. Therefore, both tested probes were considered specific and suitable for fungal detection.

The signal emitted by HRP-labeled (*P. brasiliensis*) was higher than the Texas Red-labeled (*P. lutzii*) probe signal because the TSA-FISH technique significantly increases the signal emission of hybridised probes after addition of Fluorescein in the final step of TSA (Speel et al. 1999, Kubota et al. 2006). Despite this signal difference, only the application of the FISH technique using a probe labeled with Texas Red showed a satisfactory signal emission of the hybridised fungal structures. However, the fluorescence incidence tends to be difficult to measure and interpret; it depends on the observer’s experience, which requires time and dedication to identify the hybridised target cells. In our experiment, we did not visualise the slides for a long time after hybridisation because no stabilisation solutions were used. Therefore, we indicate the use of any anti-fade reagent for longer microscopic analysis in such cases (Longin et al. 1993, Wu & Luo 2006, Fouquet et al. 2015).

The *in situ* hybridisation technique showed to be an alternative to the molecular detection of *Paracoccidioides* species by PCR and its variants, such as Nested PCR. Besides, this technique also distinguishes between *P. brasiliensis* and *P. lutzii*. In fact, the FISH or TSA-fish technique implemented here has been successfully used for detection of *P. brasiliensis* and *P. lutzii* in armadillo burrows, as demonstrated in our recent study (Arantes et al. 2016). Furthermore, the detection and differentiation of *P. brasiliensis* and *P. lutzii*, directly in the analysed sample make the *in situ* hybridisation technique a potential tool for clinical diagnosis as well. In addition to the great specificity, this approach maintains the classic diagnostic standard reference for any mycosis, which consists in displaying the fungal agent in the biological sample. Moreover, it also has the potential to monitoring the clinical evolution of paracoccidioidomycosis, as well as to assist researches on clinical and antifungal response differences between *P. brasiliensis* and *P. lutzii* during infection.
