# Supplementary material for: Use of fluorescent oligonucleotide probes for differentiation between Paracoccidioides brasiliensis and Paracoccidioides lutzii in yeast and mycelial phase
Source: Mem Inst Oswaldo Cruz. 2017 Feb;112(2):140–5. doi: 10.1590/0074-02760160374 (PMC5293123; doi:10.1590/0074-02760160374)
Supplement: Supplementary file 1 [file 0074-0276-mioc-112-2-0140-suppl01.pdf]

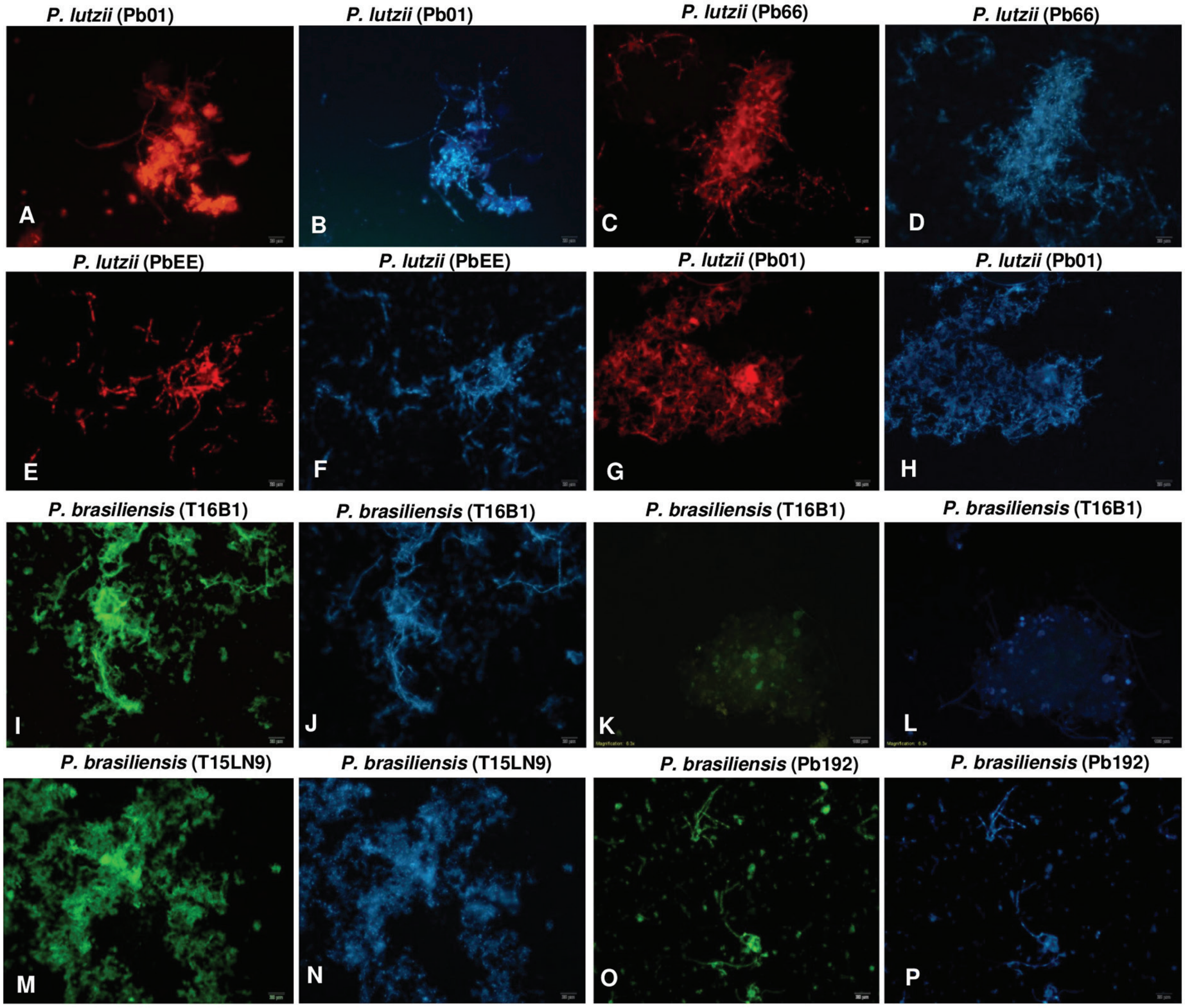

Fig. 1: FISH for *Paracoccidioides lutzii* and TSA-FISH for *P. brasiliensis* mycelia and yeast cells. (A, B, C, D) *P. lutzii* hybridised with Texas-Red probe by FISH technique; (E, F, G, H) *P. lutzii* with DAPI visualised in WU filter (Magnification 400x); (I, J, K, L) *P. brasiliensis* hybridised with HRP-Fluorescein probe by TSA-FISH technique; (M, N, O, P) *P. brasiliensis* with DAPI visualised in WU filter (Magnification 400x).

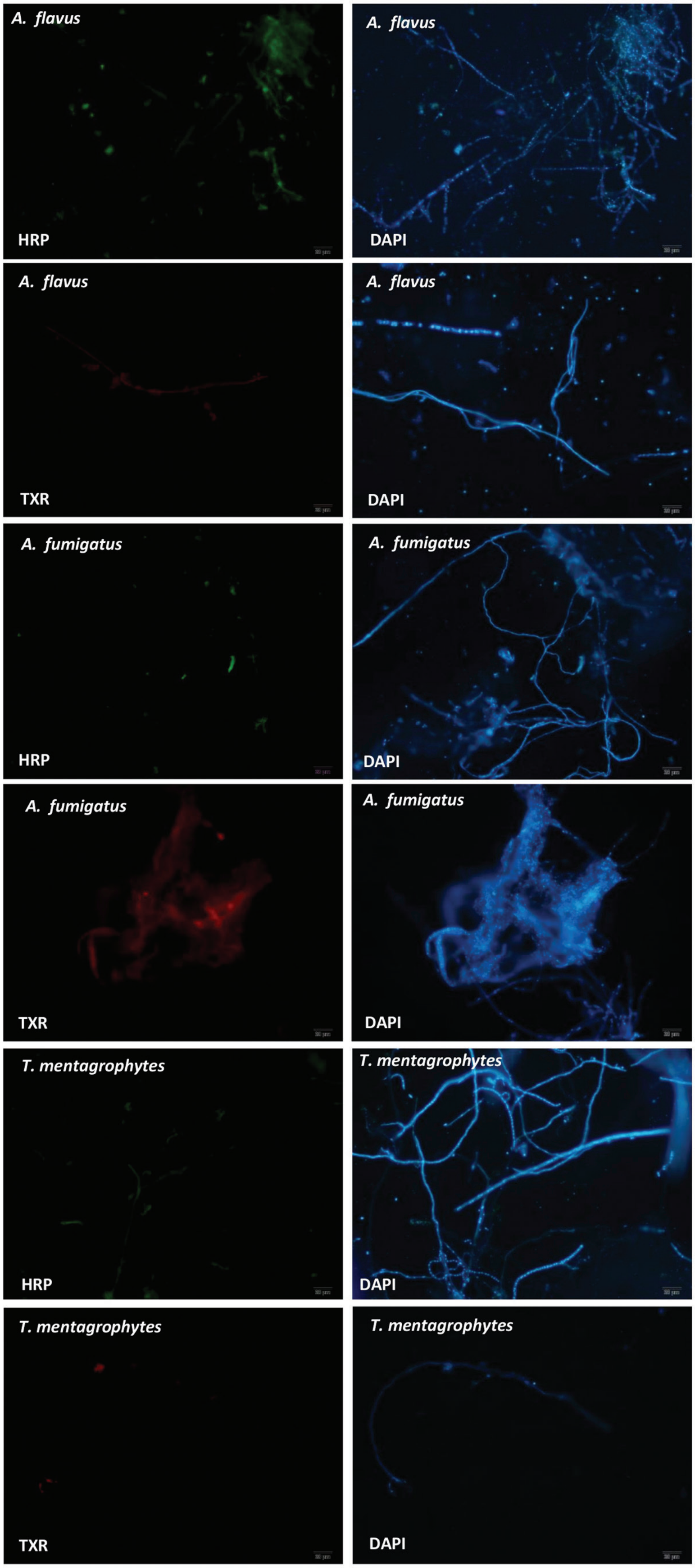

Fig. 2: specificity test in vitro. Specificity test of FISH technique (using ITS Texas Red-probe for *Paracoccidioides lutzii*) and TSA-FISH (using ITS HRP-probe for *P. brasiliensis*) against *Aspergillus flavus*; *A. fumigatus* and *Trichophyton mentagrophytes* (400x). No points of hybridised cells are present in the WG filter (Texas Red probe) and in WB filter (HRP probe). The genetic cell material is visualised with DAPI (blue) in WU filter, under fluorescence microscope.
